# Systematic Comparison of Graphene Materials for Supercapacitor Electrodes

**DOI:** 10.1002/open.201900004

**Published:** 2019-04-02

**Authors:** Lewis W. Le Fevre, Jianyun Cao, Ian A. Kinloch, Andrew J. Forsyth, Robert A. W. Dryfe

**Affiliations:** ^1^ School of Electronic and Electrical Engineering University of Manchester Sackville Street, Manchester M13 9PL UK; ^2^ School of Chemistry University of Manchester Oxford Rd Manchester M13 9PL UK; ^3^ National Graphene Institute University of Manchester Booth Street East Manchester M13 9PL UK; ^4^ School of Materials University of Manchester Oxford Rd Manchester M13 9PL UK

**Keywords:** aqueous, graphene, graphene oxide, reduced graphene oxide, supercapacitors

## Abstract

A comparison of the performance of graphene‐based supercapacitors is difficult, owing to the variety of production methods used to prepare the materials. To the best of our knowledge, there has been no systematic investigation into the effect of the graphene production method on the supercapacitor performance. In this work, we compare graphene produced through several routes. This includes anodic and cathodic electrochemically exfoliated graphene, liquid phase exfoliated graphene, graphene oxide, reduced graphene oxide, and graphene nanoribbons. Graphene oxide exhibited the highest capacitance of approximately 154 F g^−1^ in 6 M KOH at 0.5 A g^−1^ attributed to oxygen functional groups giving an additional pseudocapacitance and preventing significant restacking; however, the capacitance retention was poor, owing to the low conductivity. In comparison, the anodic electrochemically exfoliated graphene exhibited a capacitance of approximately 44 F g^−1^, the highest of the ‘pure’ graphene materials, which all exhibited superior capacitance retention, owing to their higher conductivity. The cyclability of all of the materials, with the exception of reduced graphene oxide (70 %), was found to be greater than 95 % after 10 000 cycles. These results highlight the importance of matching the graphene production method with a specific application; for example, graphene oxide and anodic electrochemically exfoliated graphene would be best suited for high energy and power applications, respectively.

## Graphical abstract

**Production matters**: Comparing the performance of graphene‐based supercapacitors in the literature is difficult, owing to the variety of testing regimes and production methods used to prepare the materials. To the best of our knowledge, there has been no systematic investigation into the effect of the graphene production method on the supercapacitor performance. In this work, graphene produced through several “standard” routes is compared. This includes anodic and cathodic electrochemically exfoliated graphene, liquid phase exfoliated graphene, graphene oxide, reduced graphene oxide, and graphene nanoribbons.

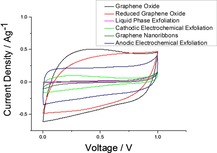

## 1 Introduction

The high power density of supercapacitors makes them particularly suitable for applications with a high peak to average power profile such as within an electric vehicle power train. A further attraction is the long cycle life, typically 100–1000 times the value for batteries, but the devices tend to have comparatively low energy density. Therefore a key focus for the rapidly growing research into supercapacitors is to combine increased energy density^1^ with a long cycle life (>100 000 cycles).^2^ Currently, supercapacitors are widely used in consumer electronics, memory backup systems and energy management.^3^ Supercapacitors can be classified as either electric double‐layer capacitors (EDLCs) or pseudocapacitors, depending on the operative charge storage mechanism. In EDLCs the capacitance arises due to the build‐up of electrolyte ions on the surface of the electrode, therefore the performance of EDLCs is dependent on the morphology of the electrode surface.^4^ Pseudocapacitors also rely on surface processes, however the capacitance now arises from very fast and reversible redox reactions which occur at the electrode‐electrolyte interface, resulting in Faradaic charge transfer.^5^ The advantages of using pseudocapacitive materials is that higher specific capacitances can be achieved than those given by EDLCs but they often suffer from performance degradation over repeated charge‐discharge cycles.^6^ However, the energy density of supercapacitors is low when compared to conventional batteries. Therefore, the focus of current research is to increase the capacitance of the devices and therefore the energy density of the supercapacitors.^1^

Porous carbons are currently used as an electrode material, with the most commonly used being activated carbon due to its moderate conductivity, high surface area, low density and high specific capacitance (150–300 Fg^−1^).^7^, ^8^, ^9^, ^10^ However, two‐dimensional (2D) materials such as graphene and transition metal dichalcogenides (TMDs) would be excellent candidates for electrochemical energy storage applications because of their high surface area and versatile electronic structure.^11^ Graphene in particular has been extensively investigated for supercapacitor applications with more than three thousand papers published on graphene‐based supercapacitors in 2017 alone. This enormous level of interest is driven by the unusual combination of useful physical properties possessed by graphene, such as high mechanical strength,^12^ specific surface area (2630 m^2^ g^−1^),^13^ thermal and electrical conductivity^14^ and theoretical maximum capacitance (quoted as 550 F g^−1^).^15^

There are various synthetic approaches to the preparation of layered 2D materials, such as graphene. For small scale device fabrication, methods such as micromechanical peeling of graphite^16^ and chemical vapour deposition^17^,^18^ are typically employed. However, for larger scale production of graphene, techniques such as liquid phase exfoliation,^19^ electrochemical exfoliation using ionic intercalation,^20^ chemical oxidation of graphite to make graphene oxide^21^ (GO), the latter sometimes followed by the reduction of graphene oxide,^22^ have been employed. These larger scale production techniques have been demonstrated in the literature and can be readily scaled up to create significant quantities of few‐layer flakes for large scale electrode production. Crucially, all these methods produce graphene with different morphologies, including differences in flake diameter and thickness, corrugation and surface chemistry.

Addition of graphene can greatly increase the performance and efficiency of supercapacitors and batteries, when used as an active electrode material or a conductive additive.^23^,^24^ Despite the extent of work on the use of graphene and its related materials in supercapacitor electrodes, there has been no direct comparison of the effect of graphene production method on supercapacitive performance. With graphene now being included in commercial supercapacitors,^25^ large‐scale production of graphene will be required. Therefore, an understanding of the influence of production method on performance is required to ensure the correct method is scaled up to produce kilogram quantities of graphene for supercapacitor applications.

Current literature on graphene‐based supercapacitors uses several different types of graphene such as liquid phase exfoliated graphene (LEG), anodic (AEEG) and cathodic (CEEG) electrochemically exfoliated graphene, graphene oxide (GO), reduced graphene oxide (rGO) and graphene nanoribbons (GNR).^13^,^26^ The reported values of specific capacitance for graphene have varied largely, depending on the testing parameters, electrolyte and electrode architecture. However, the type of graphene material used also results in large differences. For example, pure GO, rGO and AEEG electrodes have reported gravimetric capacitances exceeding 170 F g^−1[21,22,27,28]^ in aqueous electrolytes. This relatively high capacitance is attributed to two factors: the pseudocapacitive contribution from the oxygen containing groups^29^ as well as the surface defects and functionalisation which prevents the restacking of the graphene sheets.^30^ By comparison, pure liquid phase and cathodic electrochemically exfoliated graphene electrodes have reported capacitances below 50 F g^−1^ (Supplementary information of ref^11^) due to restacking of the graphene sheets, which reduces the active surface area.^31^ Therefore, several methods have been employed to increase the specific capacitance to values >100 F g^−1^ such as chemical activation^32^ or surface functionalisation.^33^,^34^

In this work we have produced five types of graphene: liquid phase exfoliated graphene (LEG), cathodic electrochemically exfoliated graphene (CEEG), anodic electrochemically exfoliated graphene (AEEG), graphene oxide (GO) using a modified Hummers method and chemically‐reduced graphene oxide (rGO) using ascorbic acid as the reductant for GO. We have also acquired graphene nanoribbons (GNR) from a commercial source, which were fabricated using oxidative unzipping of carbon nanotubes. The graphene materials were produced by “standard” conditions of the production method to give typical materials e. g. the rGO was fully reduced. These materials were first characterised by a variety of techniques including Raman spectroscopy, X‐ray photoelectron spectroscopy (XPS), nitrogen gas adsorption and scanning electron microscopy (SEM) to determine the quality of the electrode materials.

Electrodes were formed through the filtration of dispersions of the materials onto polyvinylidene fluoride (PVDF) filters to make thin membranes. This made it possible to stack the membranes back to back using the PVDF filter as the porous separator. These were then characterised electrochemically in a symmetrical two electrode CR2032 coin cell configuration using an aqueous electrolyte. This approach to electrode preparation removes the need for binding agents or conductive additives, thereby simplifying the device design and enabling the intrinsic properties of the graphene materials to be assessed. It has been shown that the use of a three electrode system can exaggerate the performance of an electrode, more than doubling the measured capacitance.^1^ Thus, the two electrode setup, using only the active material, was used as it enables accurate testing of the performance of the electrode material.

## 2 Results and Discussion

### 2.1 Characterisation of Electrode Membranes

**Figure 1:**
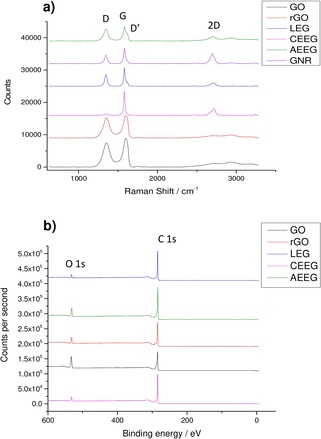
**a)** Raman spectra of all the graphene materials supported on the PVDF filter paper. The main Raman peaks of the materials are labelled. **b)** XPS of the graphene materials showing the carbon 1 s and the oxygen 1 s peaks. The relative intensity of the peaks was used to calculate the composition of the samples.

Figure 1 shows the Raman and XPS spectra for each of the graphene electrodes after the dispersions had been filtered.

Typically, for monolayer graphene, the 2D band contains one peak contribution, which is blue shifted from ∼2750 cm^−1^ (the value in bulk graphite) to ∼2690 cm^−1^, and is approximately four times more intense than the G peak.^35^ The intensity ratio of the D and G peaks, as well as the presence of the D’ peak, can also give insights into the defect concentration within the material.^36^ In the case of the LEG, GNR and CEEG the 2D peak for both materials is at ∼2720 cm^−1^ and has multiple contributions indicating the presence of multi layered flakes (>5). As both production methods used have been shown to produce few layer (1–3) flakes in dispersion, it means that restacking of the materials upon filtration has occurred. The D and D’ band in graphene is caused by the presence of edges and defects within the sample. Therefore, the LEG, AEEG and GNR spectra indicate that the flakes contain many defects due to either the ultrasonic exfoliation or oxidation. However, in the case of CEEG there is no D’ band present and the D band has a very small intensity indicating that the CEEG material is much less defective than the LEG, AEEG and GNR.

In the case of the GO and rGO, the D and G bands are shifted to higher wavenumbers due to the disorder introduced by defects and surface functionalisation. The GO spectrum contains a large D band again due to defects, which in this case are the sp^3^ hybridised carbon bound to the oxygen‐containing groups. Upon reduction of the GO, a majority of the oxygen‐containing groups are replaced with hydrogen. This also results in a large D band in the rGO spectrum as the sp^3^ hybridised carbon defect is still present.^37^

To investigate the chemical state of the graphene materials further, XPS was performed on the graphene material membranes (Figure 1b). XPS can be used to determine the oxygen concentration of the graphene materials. This is important as it has been suggested that a pseudo‐capacitive contribution arises from the oxygen groups present on the graphene surface.^21^

The only elements detected within the materials were carbon and oxygen as expected. In the case of CEEG and LEG the oxygen concentration is low (<5 %), this is expected as the two production methods are not oxidative.^19^,^38^, ^39^, ^40^ For rGO the oxygen content is low because a majority of the oxygen groups have been removed from the starting GO by the reducing agent.^22^,^41^ The oxygen content of the GO and AEEG materials are higher as both production methods are oxidative.^20^,^42^,^43^ Table 1 shows the carbon and oxygen composition of the graphene membranes calculated from the XPS survey scans.

**Table 1:** Table showing the carbon and oxygen composition for each graphene material membrane. The values for the GNR were obtained from the commercial provider.

Material	Carbon [at.%]	Oxygen [at.%]
GO	81.9	18.1
rGO	94.7	5.3
CEEG	96.1	3.9
AEEG	90.4	9.6
LEG	97.7	2.3
GNR	98.1	1.9

**Figure 2:**
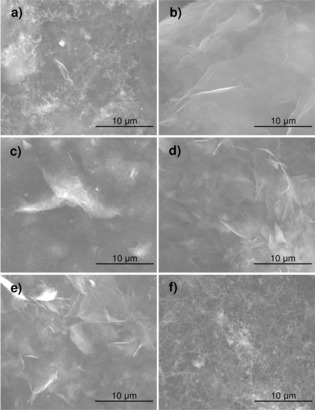
Typical SEM images of the filtered graphene membranes a) GO, b) rGO, c) LEG, d) CEEG, e) AEEG and f) GNR. The scale bar in each image is 10 μm.

To further investigate the morphology of the graphene membranes, SEM analysis was performed. Typical SEM images of each membrane are displayed in Figure 2.

Figure 2a shows the GO membrane, it consists of large (>30 μm) sheets which has resulted in a highly wrinkled surface as can be seen. Figure 2b shows the filtered rGO membrane, large uniformly stacked sheets can be seen which results in a smooth surface morphology. Figure 2c, 2d and 2e show the LEG, CEEG and AEEG membranes respectively, it can be seen that they consist of 4–10 μm sheets which have randomly re‐stacked, resulting in a highly wrinkled surface morphology. Figure 2f shows the GNR membrane: it can be seen that the GNRs have formed very densely packed random bundles, similar to that observed in CNT membranes.^44^

Finally the surface area of the electrodes was investigated using nitrogen gas adsorption. Table 2 shows the specific surface area (SSA) measured for the graphene material membranes.

**Table 2:** Table showing the specific surface areas for each graphene material membrane measured using nitrogen gas adsorption.

Material	SSA [m^2^g^−1^]
GO	759±198
rGO	669±113
CEEG	392±98
AEEG	546±134
LEG	341±135
GNR	378±151

It can be seen from table 2 that the SSA of the graphene membranes is significantly lower than the theoretical maximum (2630 m^2^ g^−1^). This is due to the restacking of the materials during electrode fabrication (filtration) which reduces the surface area. Restacking during electrode formation has been demonstrated previously for a wide range of 2D materials.^11^,^45^,^46^ The reduction in surface area due to the restacking of graphene flakes is one of the main factors for measured capacitance values lower than the theoretical maximum (550 F g^−1^). However, it can also be seen within table 3 that the equivalent series resistance (ESR) of the materials increases with increasing defect density and oxygen functionalization, (*vide infra*). Therefore, there is a trade‐off between increasing the surface area of the graphene material through the introduction of defects and increasing the conductivity through the removal of defects. This demonstrates the care needed in ensuring that the graphene material employed is suitable for the specific application.

**Table 3:** Table showing the ESR of each of the graphene membranes before and after 10,000 GCD cycles measured using EIS.

Material	Before Cycling	After Cycling
	ESR / Ω	ESR/Ω
GO	2.5±0.2	1.6±0.2
rGO	1.1±0.1	7.2±0.6
LEG	0.9±0.1	0.9±0.1
CEEG	0.3±0.1	0.3±0.1
AEEG	0.7±0.1	0.5±0.1
GNR	0.6±0.1	0.4±0.1

### 2.2 Electrochemistry

**Figure 3:**
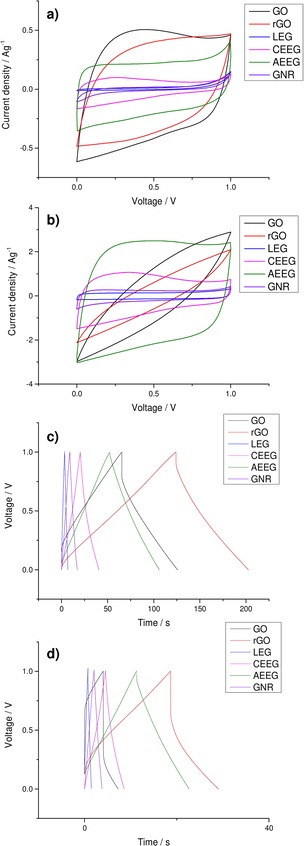
Comparison of the CVs of all the graphene materials at two different scan rates **a)** 10 mV s^−1^ and **b)** 100 mV s^−1^ and of the GCDs at two different current densities **c)** 0.5 A g^−1^ and **d)** 2 A g^−1^ before cycling.

After physical characterisation of the membranes was complete, electrochemical testing was carried out in a symmetrical coin cell. The electrolyte used was 6 M KOH: this limits the operating potential of the supercapacitor to 1 V due to the electrolysis of water at this pH.^1^ The use of organic electrolytes, such as 1 M tetraethylammonium tetrafluoroborate (TEABF_4_) in acetonitrile, allows a larger operating potential to be used, of ∼2.7 V. However, organic electrolytes have some limitations such as low conductivity and lower solubility, as well as their sensitivity to moisture. Figure 3 compares the cyclic voltammograms (CVs) and discharge curves of all the graphene material membranes at two different scan rates and current densities. Further electrochemical characterisation is provided within the supplementary information.

It can be seen that at low scan rates and current densities all the graphene materials exhibit the response typical of an EDLC. Treating Faradaic processes as pseudocapacitive, the area enclosed by the CV curve is proportional to the capacitance. Therefore, when all the materials are compared, it can be seen that GO, AEEG and rGO show significantly increased performance over the other materials. This large difference between the performance of the AEEG, GO and rGO and the other graphene materials is also seen in the galvanostatic charge‐discharge (GCD) response, as the gradient of the slope after the initial decay due to iR drop is lower for the AEEG, GO and rGO materials. However, it can be seen that when the scan rate and current density were increased both rGO and GO showed a large drop in performance (∼50–60 %). This loss in performance is attributed to the high resistance of the GO and rGO caused by the large number of defects and the oxygen functionalities present. Furthermore, it was noted that the coulombic efficiency of the rGO material was lower (60 %) when compared to the graphene materials (>95 %). The ESR of the materials was measured using electrochemical impedance spectroscopy (EIS), which was fitted using a standard Randles circuit to obtain the ESR of the materials and the results are displayed in Table 3.

This high resistance limitation has been encountered for many different materials, such as MoS_2_ or MnO_2_,^26^ and is usually solved through the addition of conductive additives such as carbon black or graphene. It has been shown that the addition of these conductive materials can increase the specific capacitance both at higher and lower discharge rates.^26^,^47^,^48^

**Figure 4:**
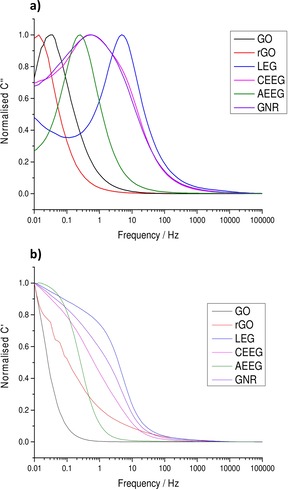
Plots of the normalised **a)** complex (C’’) and **b)** real (C’) capacitance as a function of frequency for each of the graphene materials before cycling.

Figure 4 shows the variation of the normalised real and complex values of capacitance against frequency for each of the graphene materials. The values were normalised to their respective maxima.

The low frequency values of C’ corresponds to the capacitance of the system during discharge, while C’’ corresponds to the irreversible energy losses of the system. Figure 4b plots the normalised C’ values against frequency for all the materials, and it can be seen that as the frequency increases the capacitance of the systems decreases sharply. However, the LEG, CEEG and the GNR retain their capacitances over a larger frequency range compared to GO and rGO, indicating that the GO and rGO will exhibit smaller capacitances when discharged at faster rates. This is in agreement with the CV and GCD results for these two materials.

Figure 4a shows the variation of the normalised C’’ values with frequency. The peak present in the system represents the highest frequency (shortest time) with which the capacitor can be discharged. The system will be dominated by resistive behaviour at frequencies higher than the peak and will act as an ideal capacitor at frequencies lower than the peak. It can be seen again that LEG, CEEG, AEEG and GNR can be discharged at faster rates than GO and rGO due to their increased conductivity. Therefore, when the GO and rGO are discharged at faster rates, much of the energy is dissipated by resistive losses resulting in a lower performance as seen in the CV and GCD results. The gravimetric capacitances calculated from the CVs and GCDs for all graphene materials at both scan rates and current densities before cycling are shown in Table 4.

**Table 4:** Table of gravimetric capacitances before and after cycling for all graphene materials calculated from both CV and GCD at two scan rates and current densities. Note for the rGO electrodes it was not possible to calculate the capacitance from the GCD after cycling as the discharge curve was dominated by the iR drop.

Material	Before Cycling			
	CV capacitance [F g^−1^]		GCD Capacitance [F g^−1^]	
	10 mV s^−1^	100 mV s^−1^	0.5 A g^−1^	2 A g^−1^
GO	164.6±3.2	59.2±2.1	153.7±1.6	10.9±0.8
rGO	137.4±3.3	41.3±1.2	119.6±2.6	49.4±1.9
LEG	14.8±0.7	13.4±0.8	14.5±0.4	12.7±0.5
CEEG	32.8±0.9	29.8±1.1	22.4±0.7	20.1±0.6
AEEG	44.0±1.4	42.8±1.9	46.7±1.0	40.8±0.7
GNR	11.4±0.5	9.7±0.5	12.4±0.6	11.5±0.9

Material	After Cycling			
	CV Capacitance [F g^−1^]		GCD Capacitance [F g^−1^]	
	10 mV s^−1^	100 mV s^−1^	0.5 A g^−1^	2 A g^−1^
GO	166.4±2.7	62.1±2.3	181.5±1.5	15.7±0.9
rGO	81.2±2.3	31.9±1.8	N/A	N/A
LEG	14.9±0.7	12.4±0.8	22.2±1.0	17.9±1.2
CEEG	29.2±1.1	26.5 ±1.0	27.9±0.6	25.9±0.8
AEEG	44.6±1.1	42.3±1.2	47.8±1.0	42.8±0.9
GNR	14.1±0.8	11.7±0.7	15.7±0.7	14.6±0.6

The cyclability of the supercapacitor electrodes is one of the most important metrics for comparison between materials. An ideal EDLC should have an extremely high cyclability with minimal degradation in performance after repeated GCD cycles. This high cyclability is due to the energy storage mechanism being purely physical with virtually no surface reactions occurring. For the GCD approach to cycling of graphene‐based electrodes, there are several factors that contribute to the slow degradation in capacitance. These include increased resistance between the flakes, collapse of the porous structure, desorption of active material from the current collector and for AEEG, GO and rGO surface reactions between the functional groups and the electrolyte. Therefore, the cyclability of the material is a key factor in determining its suitability for commercial supercapacitor applications. The capacitance loss for graphene including rGO and GO over 10,000 cycles can range from 30 % to no loss depending on the testing conditions.^21^,^49^

**Figure 5:**
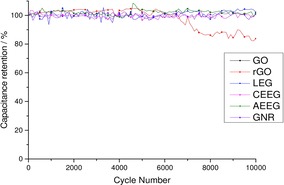
Plot of the specific capacitance retention with continued GCD at a current density of 1 Ag^−1^ for all the graphene materials over 10 000 cycles.

Figure 5 shows the cycling stability for all graphene material membranes over 10,000 GCD cycles at a current density of 1 Ag^−1^. A pre‐treatment regime of ∼100 cycles at 1 A g^−1^ was applied to all cells to ensure that the electrolyte had permeated the porous structure of the electrode.^1^

In the case of the LEG, CEEG, AEEG and GNR there was no observable degradation in performance of the material over the 10,000 cycles. This cyclability behaviour is indicative of EDLCs suggesting that this is the primary energy storage mechanism for these materials even after cycling. For GO there was a slight increase in capacitance by ∼4–5 % after 10,000 cycles. This slight increase in capacitance has been observed before and is ascribed to the gradual exfoliation of the GO electrode by the water during cycling.^21^ These measurements indicate that the GO, LEG, CEEG, AEEG and GNR show good enough cyclability to be applied in commercial supercapacitors. However, rGO showed no degradation for the first 5000 cycles but for the second 5000 a ∼20 % decrease in capacitance was observed. This loss of capacitance is to be expected due the material's poor coulombic efficiency indicating that degradation reactions are occurring. This loss in capacitance suggests that there has been a significant change in the rGO such as delamination of the electrodes. After this cycling regime, the materials were subject to further electrochemical characterisation using CV, GCD and EIS.

**Figure 6:**
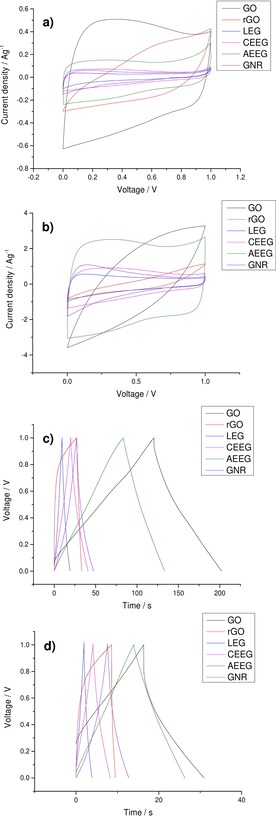
Comparison of the CVs of all the graphene materials at two different scan rates **a)** 10 mVs^−1^ and **b)** 100 mVs^−1^ and of the GCDs of all the graphene materials at two different current densities **c)** 0.5 A g^−1^ and **d)** 2 A g^−1^ after 10,000 cycles at 1 A g^−1^.

Figure 6 compares the CVs and GCDs of all the graphene material membranes at two different scan rates and current densities after 10,000 GCD cycles at 1 A g^−1^.

It can be seen in Figure 6 that for the LEG, CEEG, AEEG and GNR electrodes there has been little change in the electrochemical properties. These materials still exhibit behaviour characteristic of EDLCs, which has been reported previously.^49^ For GO, the CV has deviated from the typical rectangular shape of an ideal capacitor indicating that there is now a larger pseudocapacitive contribution to the performance.^21^ In the case of rGO it was observed that there had been significant degradation in the performance with the CV no longer showing ideal capacitor behaviour. The appearance of the CV reflects the change in the rGO electrodes which is responsible for the altered capacitive response. It can also be seen in the GCD that the resistance of the rGO has increased significantly with the discharge curve being dominated by the iR drop at all current densities. To further characterise the electrochemical behaviour of the cells, EIS was performed on the cycled cells, yielding the ESR values shown in Table 3.

**Figure 7:**
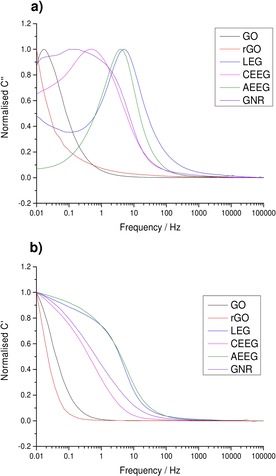
Plots of the normalised **a)** complex (C’’) and **b)** real (C’) capacitance as a function of frequency for each of the graphene materials after 10,000 CDs cycles at 1 A g^−1^.

The normalised real and imaginary components of the capacitance were also calculated, as described above, to give further insight into the processes occurring at the electrode‐electrolyte interface. These normalised values of the capacitance are shown in Figure 7. The values were normalised to their respective maxima.

After cycling, the normalised C’ value for the LEG, CEEG, AEEG, GNR and GO materials did not show any noticeable change, indicating that the capacitor response with discharge time had not changed dramatically. In the case of the rGO a sharp drop off in the C’ value was observed corresponding to the material no longer acting as an ideal capacitor for any of the measured frequencies. This is consistent with the CV and GCD results which also show a deviation from EDLC behaviour for rGO after cycling. Figure 7a shows the change in normalised C’’ values against frequency. It can be seen again that for the LEG, CEEG, AEEG, GNR and GO electrodes the peak position has remained constant indicating no change in the charge storage mechanism.

For rGO no peak was present in Figure 7a, this indicates that any current passed through the material will be completely dissipated by resistive losses resulting in no storage of energy. This result is consistent with the GCD curves obtained, as the rGO material discharge after cycling was dominated by the iR drop for all current densities used. This further indicates that significant degradation has occurred during cycling.

The gravimetric capacitances calculated from the CVs and GCDs after cycling for all graphene materials at the high and low scan rates and current densities are shown in Table 4. In every case except for the rGO device there has been a slight increase in the capacitance of the system after cycling. This behaviour has been seen before for graphene and other 2D materials, and is due to electroactivation of the electrodes. It is attributed to the partial exfoliation of the materials due to continued ion intercalation/de‐intercalation which increases the surface area and hence the capacitance.^11^,^50^,^51^

To investigate the large loss of capacitive performance of the rGO material, the cycled coin cells were opened to allow inspection of the rGO electrodes. In all cases it was found that the rGO electrodes had mechanically failed, resulting in delamination from the current collector and PVDF support. This explains the large increase in resistance as the internal wiring of the rGO electrodes had been compromised, shown in figure S1. It also explains the loss of capacitance, asymmetry in the CV and poor coulombic efficiency shown by the rGO as the amount of active material and surface area present on each electrode has been reduced. This would result in the potentials of each electrode no longer being symmetrical during operation due to the loss of mass on the positive electrode. This means that the positive electrode would reach higher potentials to equalise the charge between the electrodes resulting in degradation reactions occurring at higher voltages due to the positive electrode potential exiting the electrochemically stable potential window of the system.^52^,^53^ This situation of severe mechanical degradation has been seen for rGO electrodes before and is attributed to partial swelling of the electrodes causing mechanical stress with cycling.^54^,^55^

Another important metric when discussing the application of supercapacitor electrodes is the self‐discharge of the active material. The self‐discharge severely limits its ability to be applied to real world situations. There are three mechanisms that cause the spontaneous decrease in voltage within a supercapacitor: Faradaic processes, leakage current and charge redistribution.^56^,^57^ The Faradaic processes that occur when using an aqueous electrolyte are thought to be oxygen reduction at the negative electrode.^58^ Leakage current is the process where charged ions or impurities in the electrolyte spontaneously migrate from each electrode, which reduces the cell potential. Charge redistribution occurs when certain areas of the electrode become unevenly charged. Therefore, when charge transfer occurs between these electrode areas it results in an overall reduction of the cell potential.

For supercapacitors the voltage decreases rapidly from the initial value and the rate decreases after that with time.^56^ The initial rapid decrease in potential is due to Faradaic processes and charge redistribution occurring at the electrodes. The second more linear decrease in potential after the initial decrease is due to leakage current.

**Figure 8:**
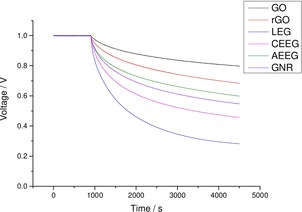
Self‐discharge behaviour for all the graphene materials after undergoing potentiostatic charging at 1 V for 15 minutes prior to measuring the open circuit voltage.

Figure 8 plots the open circuit voltage of each of the graphene materials (pre‐cycling) after undergoing 15 minutes of potentiostatic charging at 1 V. The open circuit voltage was then measured for 1 hour.

It can be seen that the GO and rGO electrodes exhibit the best charge retention with the pure graphene materials (AEEG, GNR, CEEG and LEG) showing progressively worse retention. This is believed to be due to the pseudocapacitive functional groups present on the surface allowing for reduced Faradaic leakage and charge redistribution across the membrane.^11^ These electrochemical results indicate that GO and AEEG display favourable properties for high energy and high power supercapacitor applications, respectively. However, the reduced conductivity of the GO means that there is a reduced performance for faster discharge times. Therefore, a conductive additive, such as carbon black, LEG or CEEG, is required to counteract this decrease in performance.

## 3 Conclusions

In summary, the electrochemical performances of six different graphene‐derived materials were compared as electrodes for supercapacitors. The materials that were tested included liquid exfoliated graphene, cathodic and anodic electrochemically exfoliated graphene, commercial graphene nanoribbons, graphene oxide and reduced graphene oxide. It was observed that graphene oxide and reduced graphene oxide showed the highest specific capacitances of ∼153 F/g and ∼119 F/g, respectively. It was also observed that the anodic electrochemically exfoliated graphene showed the highest capacitance, ∼44 F/g, of the pure graphene samples. The increased capacitance in these materials is due to the oxygen containing functional groups present on the materials imparting an additional pseudo‐capacitive contribution. However, the reduced graphene oxide electrodes underwent a severe degradation in performance and mechanically failed during galvanostatic charge discharge cycling. The other graphene materials showed no loss in performance after cycling. However, due to the decreased conductivity caused by the presence of the oxygen groups, the graphene oxide electrodes showed a large loss in performance for faster discharge rates due to resistive losses. This can be overcome through the addition of conductive additives such as graphene or CNTs. For the other graphene materials, very little loss in capacitance was observed for increased discharge rates. For high energy applications involving graphene materials, graphene oxide would be the material of choice whereas for high power applications involving graphene, anodic electrochemically exfoliated graphene would be the best choice. These electrochemical results suggest that the graphene morphology and hence production method plays a large role in its supercapacitive performance. Therefore, the possible supercapacitor application should dictate the type of graphene used.

## **Experimental Section**

### Preparation of Liquid Exfoliated Graphene

Graphene dispersions were created by a liquid phase exfoliation method that has been reported previously.^19^,^40^ Briefly, flake graphite (Sigma Aldrich) was dispersed in N‐methyl‐2‐pyrrolidone (NMP) (10 mgml^−1^) and ultrasonically processed in a bath sonicator (Elmasonic P70H). The bath sonicator was operated at 37 kHz and 40 % amplitude for 12 hours with cooling to maintain a stable temperature of 20 °C. The sonicated dispersions were centrifuged at 6000 rpm (3139 g) for 30 minutes to remove any unexfoliated material. The supernatant was decanted and fresh solvent was added before repeating the centrifugation so that a narrow distribution of flake dimensions and thicknesses was obtained. These graphene dispersions were stable in solution for several months with virtually no detectable sedimentation. The yield of few‐layer LEG flakes for this technique is typically ∼2 %.

### Preparation of Cathodic Electrochemical Exfoliated Graphene

Graphene dispersions were created using a modified electrochemical exfoliation method that has been reported previously.^59^ Briefly, 30 grams of LiCl and triethylamine hydrochloride were dissolved into 1 L of dimethyl sulfoxide (DMSO). Flake graphite (Sigma Aldrich) and PVDF (5 wt. %) were then mixed together in ethanol to make a slurry which was dried in a vacuum oven overnight at room temperature. The dry powder was pressed into pellets and sintered at 450 °C under argon. Then the pellet was used as the working electrode and graphite source for the exfoliation. The pellet, platinum mesh counter electrode and Ag/AgCl reference electrode, were placed into the DMSO solution and the potential was then held at −20 V for 48 hours. The exfoliation products were filtered and then washed with water and ethanol to remove any residual exfoliating ions. Products were dried at 200 °C under an argon atmosphere, with the resulting dry powder being re‐dispersed in NMP before bath sonication (Elmasonic P70H) at 37 kHz and 40 % amplitude for 30 minutes to ensure a homogenous suspension. Dispersions of the CEEG were centrifuged at 500 rpm (21 g) for 30 minutes to remove any unexfoliated material. These graphene dispersions were stable in solution for several months with virtually no detectable sedimentation. The yield of few layer CEEG flakes for this method is typically ∼1–2 %.

### Preparation of Anodic Electrochemical Exfoliated Graphene

Graphene dispersions were created using an anodic electrochemical exfoliation method.^60^ Briefly, a graphite foil working electrode and platinum mesh counter electrode were placed into a 0.1 M (NH_4_)_2_SO_4_ in water and a potential of 10 V was applied for 2 hours. After the graphite exfoliation was completed, the product was washed several times with deionised water by vacuum filtration. The resultant graphene was then dispersed in N,N’‐dimethylformamide (DMF) by sonication (Elmasonic P70H) at 30 % amplitude for 30 minutes. The dispersion was left for 48 hours to precipitate any un‐exfoliated graphite flakes or particles. The top part of the dispersion was filtered and washed twice with water. The filter cake was then freeze‐dried to obtain electrochemically exfoliated graphene. The yield of few layer AEEG flakes from this method is typically ∼30–40 % and the oxygen content for these flakes was ∼9 % determined by XPS.

### Preparation of Graphene Oxide

Graphene oxide was produced using a modified Hummers method.^43^ Briefly, 65 ml of sulphuric acid was added to a mixture of 5 grams of natural graphite (Sigma Aldrich) and 2.5 grams of sodium nitrate in an ice bath with stirring. Then 15 grams of potassium permanganate was slowly added and left to stir overnight. The mixture was diluted to 1 L and 10–20 ml of 30 % hydrogen peroxide was added slowly to quench excess potassium permanganate, the solution was then left stirring for 2 hours. This solution was filtered and first washed with 10 % HCl to remove excess metal ions and then with water to reach a pH of 7. The products were re‐dispersed in NMP and sonicated (Elmasonic P70H) at 37 kHz and 40 % amplitude for 2 h to exfoliate the GO to a few layers. These dispersions then went through the same centrifugation process as the LEG to remove any unexfoliated material. These GO dispersions were stable in water for several months with virtually no detectable sedimentation. The yield of few‐layer GO flakes for this method is typically ∼90 % and the oxygen content for these flakes was ∼20 %, as determined by XPS.

### Preparation of Reduced Graphene Oxide

The chemical reduction of GO was done using a method that had been previously reported.^41^ To summarise: a GO dispersion was made using the aforementioned GO powder with a concentration of 0.1 mg/ml. Ascorbic acid (vitamin C) was added to the GO dispersion to give a concentration of 0.1 M. Then approximately 5 ml of ammonia was added to the GO solution to increase the pH of the system to 10. The reaction mixture was heated to 95 °C with stirring and left to react for 4 hours. The resultant rGO was filtered and washed with water to remove the ascorbic acid and to reach a pH of 7. The material was then re‐dispersed in NMP and sonicated, as performed with the GO, to exfoliate the rGO to a few layers. The dispersions underwent the same centrifugation treatment as the previous materials to ensure that no unexfoliated material remained. The rGO dispersions were stable in NMP for several months with virtually no sedimentation. The yield of few layer rGO flakes for this method is typically close to 100 %.

### Preparation of Graphene Nanoribbons

Commercially sourced GNR powders were dispersed in NMP (10 mg/ml) before sonicating (Elmasonic P70H) at 37 kHz and 40 % amplitude for 30 minutes to ensure a homogenous suspension. This GNR dispersion was put through the same centrifugation treatment as the liquid phase exfoliated graphene material to ensure that no exfoliated material remained. These GNR dispersions were stable in solution for several months with virtually no detectable sedimentation.

### Preparation of Graphene Material Membranes

Membranes of the graphene materials were created by first diluting the NMP solutions (15‐fold dilution) with isopropanol (IPA). These diluted solutions were filtered through preweighed PVDF filters (Millipore) with a 0.1 μm pore size. The filtered membranes were then washed with IPA to remove any residual NMP and dried in an oven overnight at 80 °C to remove any residual solvent. The mass of the graphene materials was measured by weighing the membrane after drying and was typically between ∼4–5 mg. The graphene membranes were analysed by Raman spectroscopy, SEM, XPS and then used as the electrodes within coin cells.

### Raman Spectroscopy

Raman spectroscopy was performed to give an initial characterisation of the filtered membranes. This technique can enable the determination of the layer number and defect density of the graphene materials after filtration. To prepare the samples for Raman analysis the filtered membranes were placed in a vacuum oven at 100 °C to ensure that all solvent had been removed. Raman measurements were performed using a Renishaw inVia Microscope with 532 nm (2.33 eV) laser excitation at a power of 1 mW with a 100× objective. A grating of 1800 mm^−1^ was used, which gave a spectral resolution of ∼1 cm^−1^.

### Scanning Electron Microscopy

Scanning electron microscopy (SEM) analysis was performed on the filtered membranes supported by the PVDF with a FEI/Phillips XL30 E‐SEM under high vacuum conditions with an accelerating voltage of 15 kV. All images were taken using the secondary electron detector.

### X‐ray Photoelectron Spectroscopy

X‐ray photoelectron spectroscopy (XPS) was performed using a Kratos Axis Ultra DLD spectrometer with a monochromated Al K_α_ X‐ray source (E=1486.6 eV, 10 mA emission), a hemispherical electron energy analyser and a multichannel plate and delay line detector (DLD). The analysis area of the membranes was 300 μm×700 μm. Survey spectra were collected to determine the elemental composition. Charge neutralisation was used for all samples.

### Nitrogen Gas Adsorption

Nitrogen gas adsorption measurements were performed on the graphene material membrane electrodes using a Quadrasorb‐evo Gas Sorption Surface Area and Pore Size Analyser (Quantachrome). To avoid the contribution of the highly porous PVDF support, the graphene material was removed from the PVDF before testing. To ensure that the measurement could be completed in the 36 hours of testing time available, approximately 30–40 mg of sample was tested during each run. The specific surface area of the materials was calculated using non localised density functional theory with a slit pore model.

### Electrochemistry

Cyclic voltammetry (CV), electrochemical impedance spectroscopy (EIS), galvanostatic charge‐discharge (GCD) and self‐discharge (SD) were performed using a PGSTAT302 N potentiostat (Metrohm Autolab). All electrochemical measurements were performed using a sealed symmetrical coin cell (CR2032) system; for each material a minimum of four coin cells were measured. The membranes were stacked back‐to‐back within the coin cell with the active material making direct contact with the stainless steel spacer, which acted as a current collector; a steel wave spring was used to ensure a good contact was kept during the testing. The electrolyte used was aqueous 6 M KOH. CV was performed at scan rates ranging from 10 to 500 mV/s. EIS was performed at a frequency range of 0.1 Hz to 100 kHz with a 10 mV (rms) perturbation and 0 V dc bias. GCD of the cells was performed at current densities ranging from 0.5 A/g to 4 A/g. The specific capacitance was calculated using the best practice methods established by Ruoff.^1^

## Conflict of interest

The authors declare no conflict of interest.

## Supporting information

As a service to our authors and readers, this journal provides supporting information supplied by the authors. Such materials are peer reviewed and may be re‐organized for online delivery, but are not copy‐edited or typeset. Technical support issues arising from supporting information (other than missing files) should be addressed to the authors.

Supplementary
