# Supplementary material for: Systematic Comparison of Graphene Materials for Supercapacitor Electrodes
Source: ChemistryOpen. 2019 Apr 2;8(4):418–28. doi: 10.1002/open.201900004 (PMC6445062; doi:10.1002/open.201900004)
Supplement: Supplementary file 1 — Supplementary [file OPEN-8-418-s001.pdf]

Supporting Information

© Copyright Wiley-VCH Verlag GmbH & Co. KGaA, 69451 Weinheim, 2019

Systematic Comparison of Graphene Materials for Supercapacitor Electrodes

Lewis W. Le Fevre, Jianyun Cao, Ian A. Kinloch, Andrew J. Forsyth,* and Robert A. W. Dryfe*© 2019 The Authors. Published by Wiley-VCH Verlag GmbH & Co. KGaA.
This is an open access article under the terms of the Creative Commons Attribution License, which permits use, distribution and reproduction in any medium, provided the original work is properly cited.

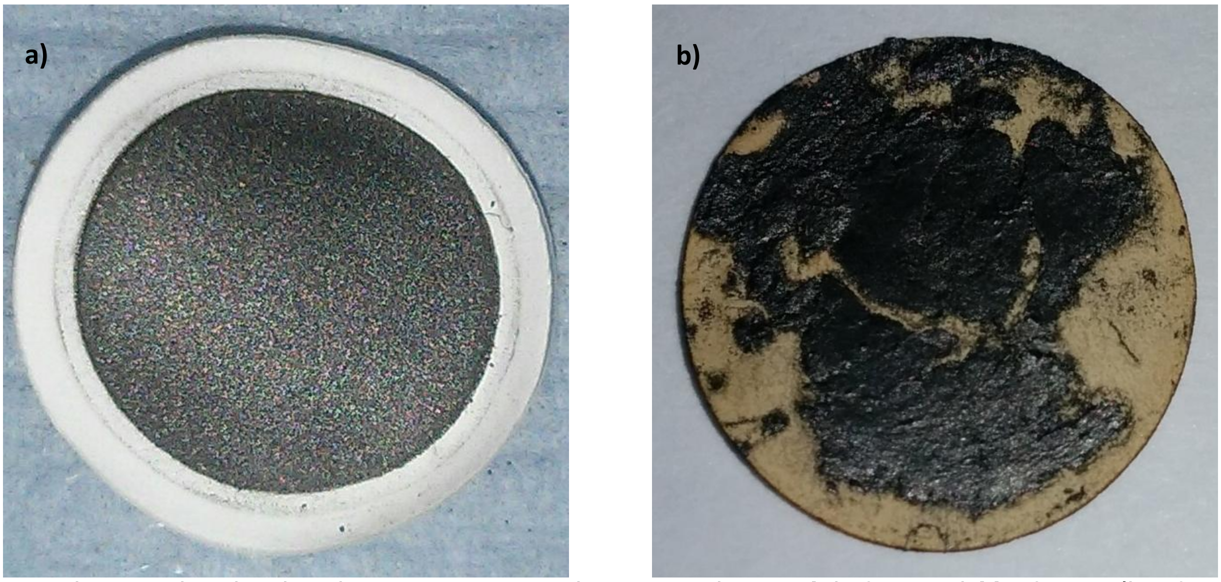

S1: Photographs showing the PVDF supported rGO membrane **a)** before and **b)** after cycling for 10,000 cycles at 1 Ag^{-1}.

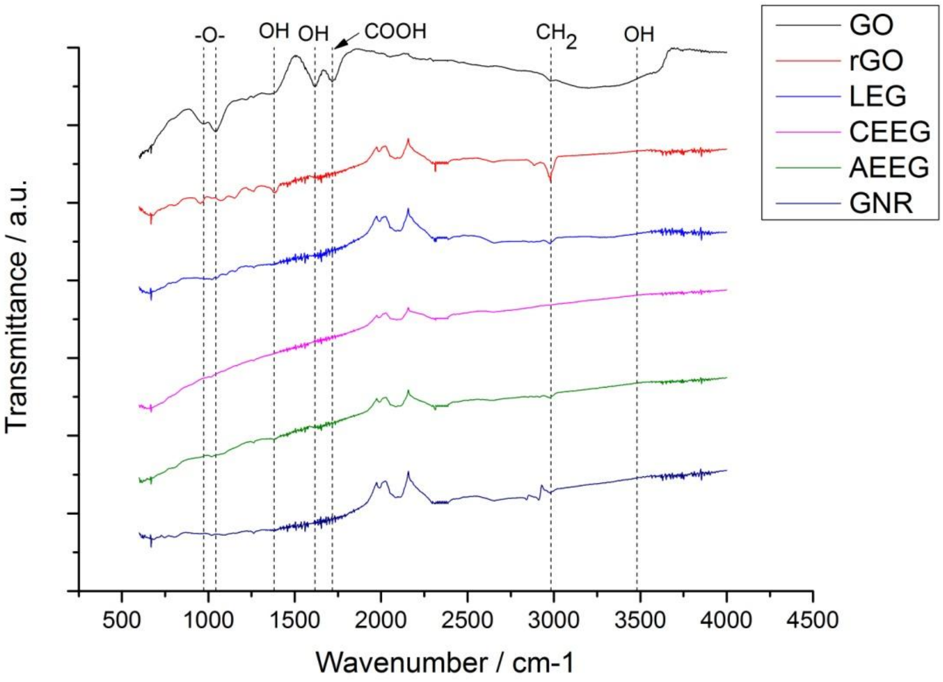

S2: Fourier Transform infra red spectra for the produced graphene materials. The black dashed lines represent the peak positions of the corresponding functional groups present within the spectra.

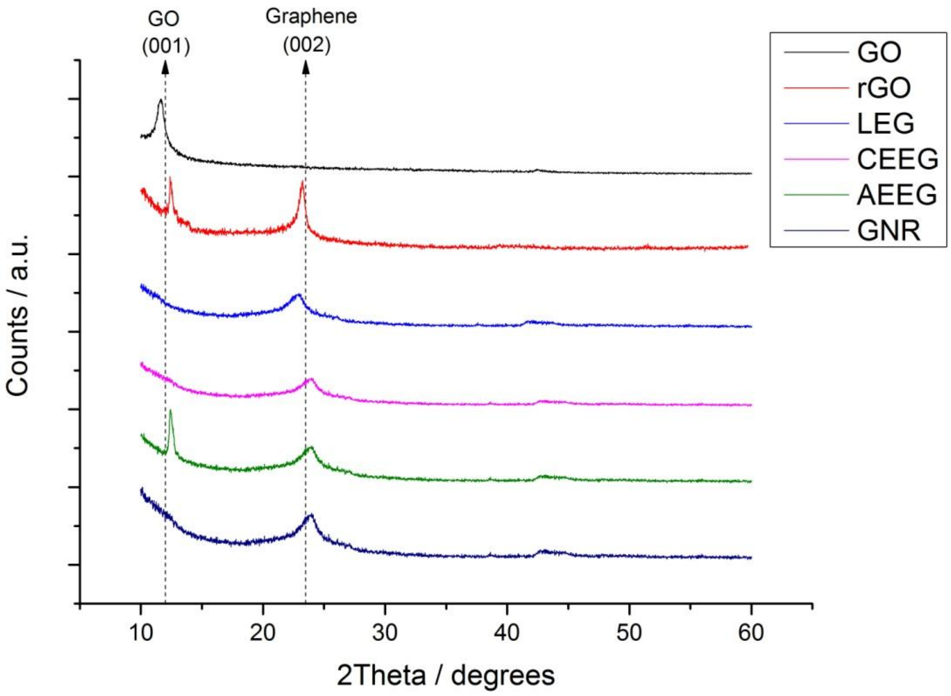

S3: X-ray diffraction spectra for each graphene material membrane produced. The peaks present within the spectra have been labelled with the associated material and miller plane.

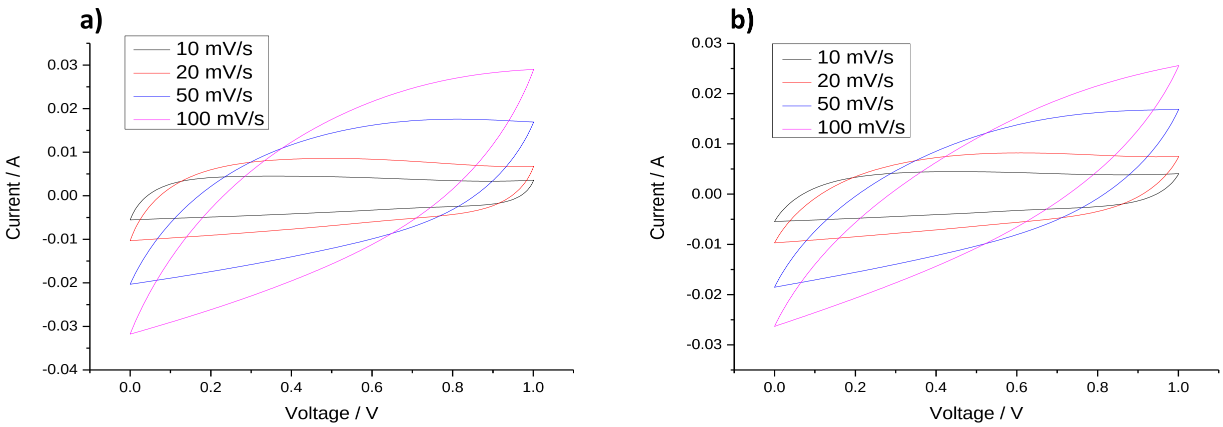

S4: Plots of the CVs with varying scan rate for the GO electrodes **a)** before and **b)** after 10,000 CDs cycles at 1 Ag^{-1}.

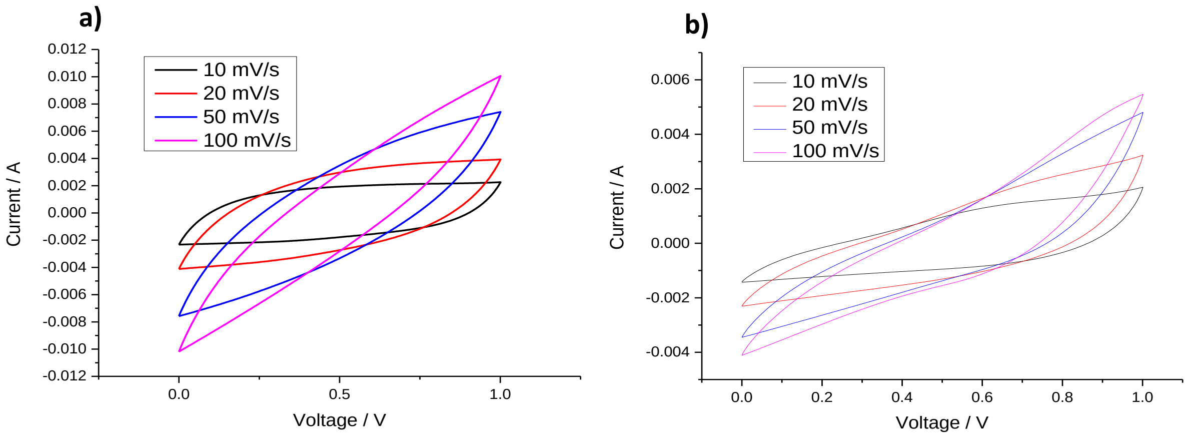

S5: Plots of the CVs with varying scan rate for the rGO electrodes **a)** before and **b)** after 10,000 CDs cycles at 1 Ag^{-1}.

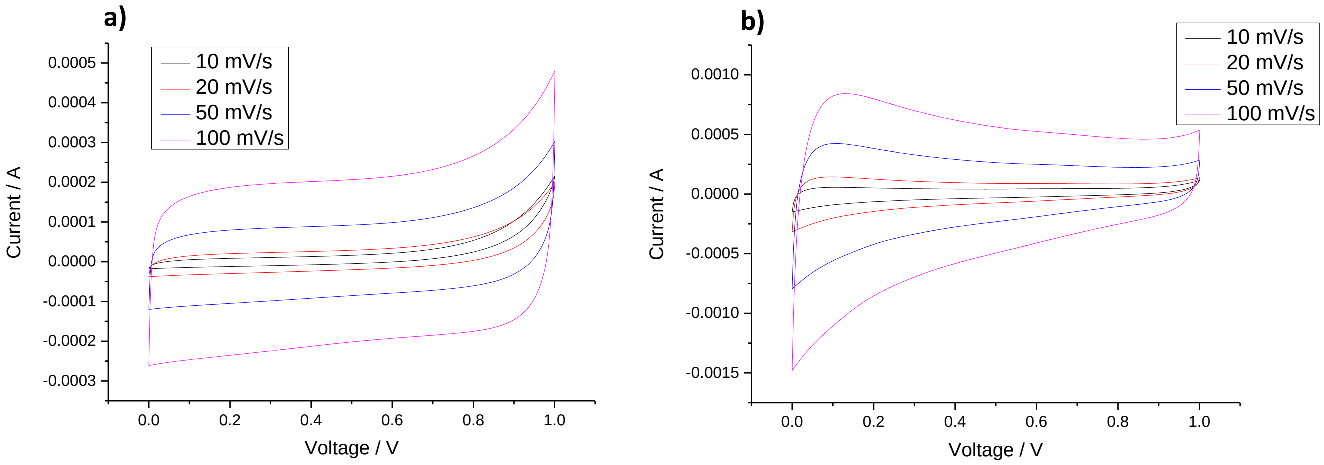

S6: Plots of the CVs with varying scan rate for the LEG electrodes **a)** before and **b)** after 10,000 CDs cycles at 1 Ag^{-1}.

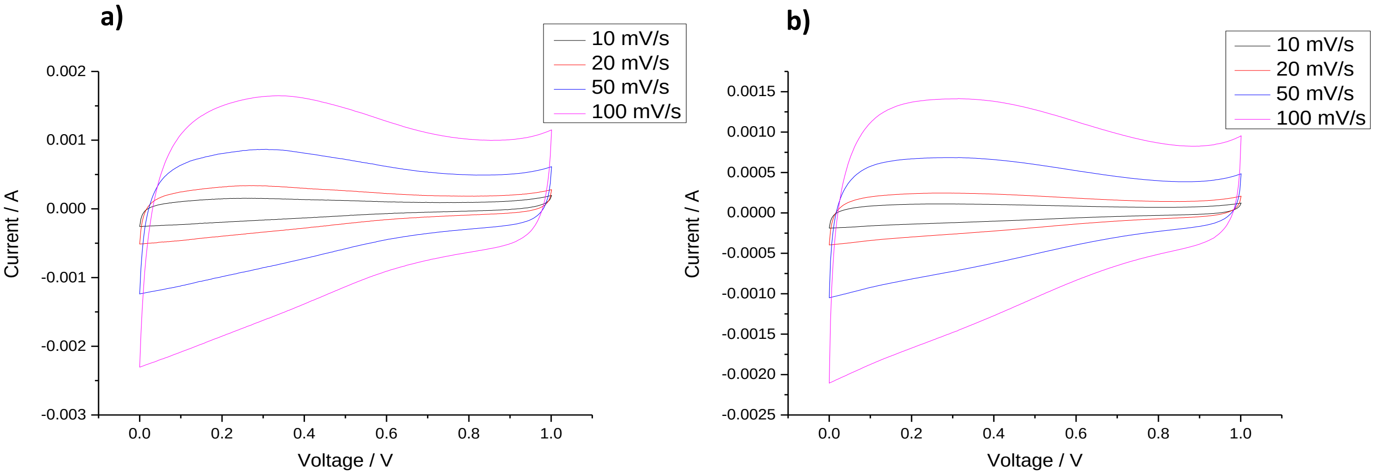

S7: Plots of the CVs with varying scan rate for the CEEG electrodes **a)** before and **b)** after 10,000 CDs cycles at 1 Ag^{-1}.

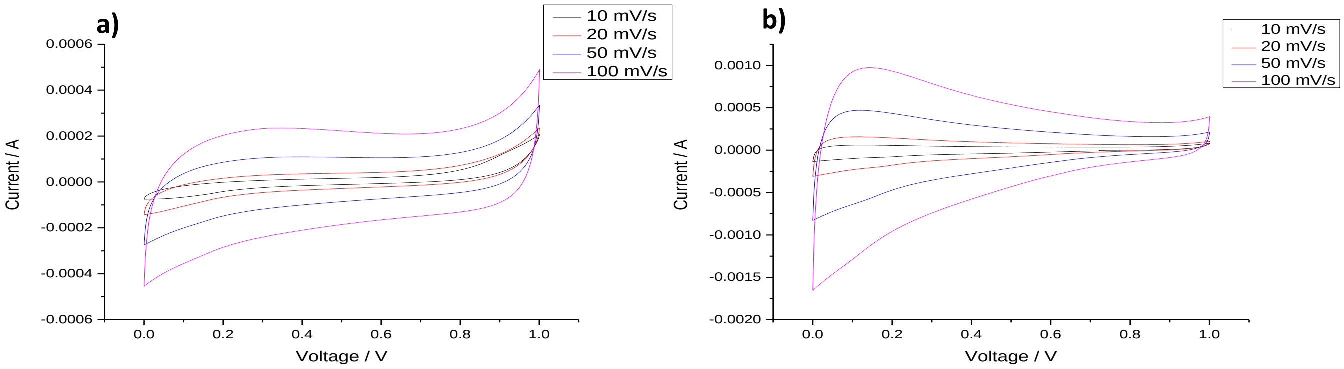

S8: Plots of the CVs with varying scan rate for the GNR electrodes **a)** before and **b)** after 10,000 CDs cycles at 1 Ag^{-1}.

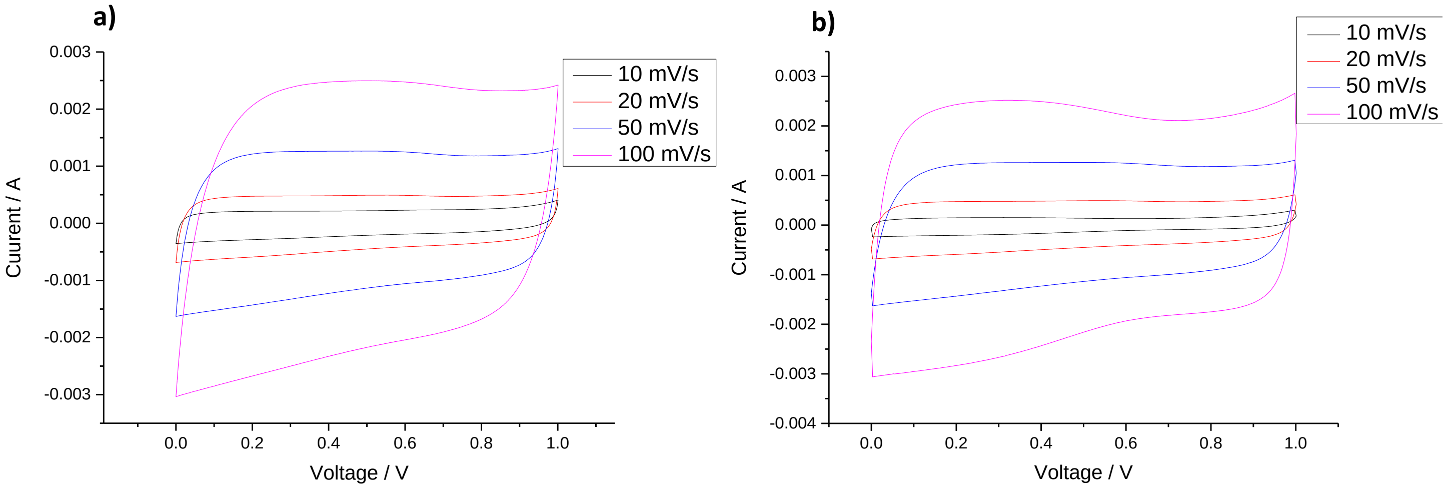

S9: Plots of the CVs with varying scan rate for the AEEG electrodes **a)** before and **b)** after 10,000 CDs cycles at 1 Ag^{-1}.

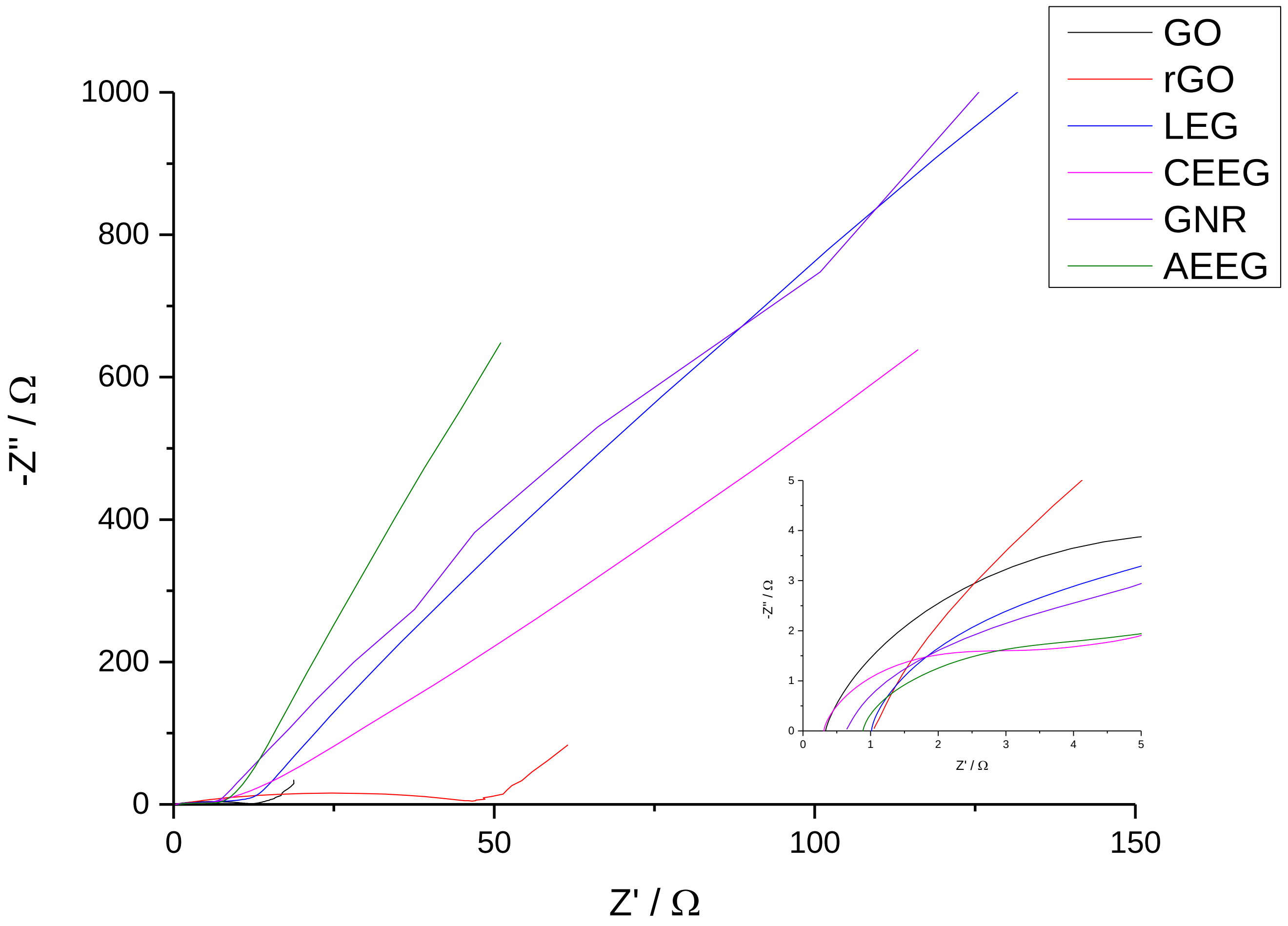

S10: Nyquist plots for all the graphene materials tested. The inset is a magnification of x intercepts for the materials before cycling.
